# Prenatal Sex Determination and Gender Bias: Awareness and Attitudes Among Pregnant Women in South India

**DOI:** 10.7759/cureus.115907

**Published:** 2026-09-07

**Authors:** Subhashree Nandy, Pracheth Raghuveer, Poonam Naik, Habeena Shaira

**Affiliations:** 1 Community Medicine, Yenepoya Medical College, Mangalore, IND; 2 Epidemiology, National Institute of Mental Health and Neurosciences, Bengaluru, IND; 3 Community Medicine, GR Medical College, Mangalore, IND

**Keywords:** antenatal women, gender preference, pcpndt act, prenatal, sex determination

## Abstract

Background and aim: There persists a strong gender preference for the male child in India, despite socioeconomic development. Pre-Conception and Pre-Natal Diagnostic Techniques (PCPNDT) Act was introduced in 2002, with the main purpose of preventing misuse of diagnostic techniques in sex selection and sex-selective abortion. The present study primarily aimed to determine the proportion of pregnant women who are aware of prenatal sex determination and the PCPNDT Act, and the proportion of women with gender preference for the unborn child among pregnant women attending an outpatient facility attached to a medical college hospital in Mangalore, South India. Secondarily, this study aimed to assess the perception regarding prenatal sex determination, the PCPNDT Act, and preference to know the sex of the unborn child among the participants, and to determine the factors associated with awareness of prenatal sex determination, the PCPNDT Act, and gender preference.

Materials and methods: This cross-sectional study was conducted among pregnant women who visited the antenatal care facility attached to a medical college hospital in Mangaluru. Convenience sampling was used to enroll participants into the study. A predesigned and pretested proforma was used to collect data via Epicollect5 (Oxford, UK: Oxford Big Data Institute, University of Oxford). Categorical variables were summarized as proportions and percentages, and multiple logistic regression was used to calculate confounder-adjusted estimates.

Results: A total of 169 antenatal women who fulfilled the eligibility criteria were enrolled in this study. The proportion of pregnant women who knew about prenatal sex determination was 42.0%, and 15.4% knew about the legislation that prevents it. A total of 36.6% of pregnant women had a gender preference for the unborn child, while 27.8% preferred to know the sex of the unborn child.

Conclusions: In this study, only a small proportion of women knew about the PCPNDT Act, while the preference for a specific gender and the willingness to know the sex of the unborn child were higher. The findings underscore the need to address the intersection of socioeconomic limitations and cultural preferences around gender preference and prenatal sex determination through targeted strategies for at-risk groups.

## Introduction

Sex ratio is regarded as an important social indicator of prevailing equity between males and females in society [1]. India has had a history of adverse female sex ratios since 2001. According to the 2011 census, the sex ratio is 933 females per 1,000 males. One of the main factors leading to this adverse sex ratio in India is sex-selective female feticide, which is preventable. Further, there persists a strong gender preference for the male child in India, despite socioeconomic development [2]. To counter this, the government of India introduced the Pre-Natal Diagnostic Techniques (PNDT) Act in 1994, which was later amended as the Pre-Conception and Pre-Natal Diagnostic Techniques (PCPNDT) Act in 2002, with the main purpose of preventing misuse of diagnostic techniques in sex selection and sex-selective abortion. Violation of this act can lead to a fine of Rs. 10,000 and up to three years' imprisonment for a first offense, with greater fines and longer terms of imprisonment for repeat offenders [3].

When compared to the national average, the sex ratio in Dakshina Kannada district, Karnataka, South India, is better, with a ratio of 1,018 females per 1,000 males. This could be due to a high female literacy rate and a matriarchal system of inheritance followed in certain communities. However, as per the latest census, the child sex ratio seems to have dropped from 952 in 2001 to 946 in 2011, which is marginally better than the average for Karnataka (943) [2].

There are quite a few studies that have assessed the awareness among pregnant women regarding prenatal sex determination and the PCPNDT Act. Among the studies done among pregnant women from Karnataka, Kumar et al. assessed awareness about prenatal sex determination, the PCPNDT Act, and preference to know the sex of the unborn child, while Kumar et al. assessed the preferred gender composition. However, limited evidence is available on studies that have simultaneously examined awareness of prenatal sex determination, knowledge of the PCPNDT Act, and the preference to know the sex of the unborn child. This may facilitate the design of appropriate interventions to improve awareness of the PCPNDT Act, particularly among antenatal women.

## Materials and methods

We conducted a cross-sectional study at the outpatient department (OPD) run by the department of obstetrics and gynecology (OBG) attached to a medical college hospital in Mangalore, Karnataka, South India. This study was carried out over a period of two months, June and July 2019.

Pregnant women who visited the hospital OPD for antenatal care during the morning hours, regardless of gestational age, were enrolled until the sample size was achieved in the study. Those who were unable to communicate with the investigators verbally were excluded.

The sample size was estimated using the formula $n = \frac{Z^2 \times p \times q}{e^2}$ [4]. Here, n is the required sample size, Z is the standard normal deviate, which is equal to 1.96 at a 5% significance level, p is the estimated prevalence, and e is the margin of error. The awareness regarding prenatal sex determination among pregnant women, p was taken as 91.4%. With an absolute precision of 5% and a non-response rate of 10%, the estimated sample size was 130 [4]. The awareness regarding PCPNDT among pregnant women (p) was reported as 68.0% (Sharmila et al., Puducherry), with a relative precision of 15% and a non-response rate of 10%; the estimated sample size was 92 [5]. Taking 39.40% (Kumar et al., Mangalore) as the prevalence of gender preference of the unborn child among the study participants (p), with a relative precision of 20% and a non-response rate of 10%, the estimated sample size (SS) was 169 [4,6].

Data were collected using a predesigned and pretested proforma through Epicollect5 (Oxford, UK: Oxford Big Data Institute, University of Oxford) to avoid data entry errors. The proforma was administered in Kannada by final-year medical students who received training and practical exercises prior to data collection. The questionnaire was translated and back-translated. The questionnaire had sections on sociodemographic characteristics, obstetric history, awareness, and perception. For this study, awareness of prenatal sex determination is considered if the pregnant women know that it is possible to determine the sex of the unborn child, and awareness of the PCPNDT Act is considered if the pregnant women know about a government act that is against prenatal sex determination. Perception towards the PCPNDT Act was studied through the following two independent outcomes: opinion on the need for punishment for prenatal sex determination and willingness to educate others about the PCPNDT Act. Other details around various aspects of prenatal sex determination and the PCPNDT Act were also included. Socioeconomic status (SES) was assessed using the modified BG Prasad Socioeconomic Classification (2019).

Data were analyzed using IBM SPSS version 23.0 (Armonk, NY: IBM Corp.) statistics for Windows by the principal investigator (PI). The proportion of pregnant women who were aware of prenatal sex determination and the PCPNDT Act, and the proportion of pregnant women who had gender preference were expressed in percentages. Proportions and percentages were used to express categorical variables such as sociodemographic and obstetric variables, variables pertaining to awareness of prenatal sex determination and the PCPNDT, and questions assessing perceptions of prenatal sex determination and the PCPNDT Act. A chi-square test was performed to detect differences between proportions, and variables having a p<0.2 in univariate analysis were considered for the final regression model. To determine the predictors of awareness about the PCPNDT Act and gender preference of the unborn child among the participants, multiple logistic regression (MLR) was applied to provide confounder-adjusted estimates of the association between the sociodemographic and obstetric variables and awareness regarding the government act against prenatal sex determination and male gender preference of the unborn child. Adjusted odds ratios (AORs) with 95% confidence intervals (95% CIs) were computed, and p<0.05 was considered statistically significant.

## Results

A total of 169 antenatal women who fulfilled the eligibility criteria participated in the present study. The non-respondents were 16 (9.47%). The mean age of the participants was 26.58±4.42 years. Around one-third of respondents were educated up to grade 7. A wide majority (94.70%) were housewives. More than half (61.50%) belonged to the third trimester. The mean gestational age of participants was 27.37±8.54 weeks (Table 1).

**Table 1:** Sociodemographic and obstetric profile of the study participants (n=169). *Socioeconomic status (SES) using modified BG Prasad Socioeconomic Classification (2019) [7].

Variables		Number	Percentage
Age (years)	18-29	123	72.80
Religion	Hindu	22	13.0
	Muslim	144	85.20
	Christian	3	1.80
Education status	No formal education	6	3.60
	Educated up to grade 7	53	31.40
	Educated up to grade 10	65	38.50
	Educated up to grade 12	29	17.20
	Graduate and above	16	9.50
Occupation	Working	9	5.30
	Housewife	160	94.70
Husband’s education status	No formal education	6	3.60
	Educated up to grade 7	57	33.70
	Educated up to grade 10	80	47.30
	Educated up to grade 12	16	9.50
	Graduate and above	10	5.90
Husband’s occupation	Working	167	98.80
	Not working	2	1.20
SES [7]*	Class I	9	5.30
	Class II	41	24.30
	Class III	45	26.60
	Class IV	45	26.60
	Class V	29	17.20
Gestational age	First trimester	17	10.10
	Second trimester	48	28.40
	Third trimester	104	61.50
Number of living children	No child	69	40.80
	One child	55	32.50
	Two children	30	17.80
	>Two children	15	8.87
Gender of the last living child	Boy	54	32.0
	Girl	46	27.0
	Not applicable	69	40.80

Nearly 71 (42.0%) respondents answered that prenatal sex determination is possible. A high percentage (84.60%) did not know about the methods of prenatal sex determination. About 63 (37.30%) believed that prenatal sex determination is a punishable offense, but more than half (62.0%) were unaware of who would be punished (Table 2). Around 15.40% of the respondents had heard of a government act against prenatal sex determination. Only one participant could mention the name of the act correctly. A total of 99 (58.60%) respondents opined that prenatal sex determination should be punished. After receiving education on the PCPNDT Act, the respondents were asked if they would disseminate the information to others [3]. In response to this question, a high percentage (76.90%) expressed willingness to educate others about the PCPNDT Act (Table 2). The proportion of pregnant women with any gender preference for the unborn child was 36.6%. When probed further, 11.20% preferred a boy child for the current pregnancy, while 25.40% preferred a girl child. Nearly 63.30% had no gender preferences (Table 2).

**Table 2:** Awareness of prenatal sex determination, awareness and perceptions of PCPNDT Act, and gender preferences among participants (n=169). *Pre-Conception and Pre-Natal Diagnostic Techniques (Prohibition of Sex Selection) Act [3]. PCPNDT: Pre-Conception and Pre-Natal Diagnostic Techniques

Variables		Number	Percentage
Awareness of prenatal sex determination			
Is prenatal sex determination possible?	Yes	71	42.0
	No	27	16.0
	Don’t know	71	42.0
Techniques	Ultrasound	26	15.40
	Don’t know	143	84.60
Place	Government hospital	2	1.20
	Private hospital	11	6.50
	Both	20	11.80
	Don’t know	136	80.50
Is prenatal sex determination punishable?	Yes	63	37.30
	No	3	1.80
	Don’t know	103	60.90
Person punished	Doctor	15	8.90
	Patient/relatives	11	6.50
	Both	35	20.70
	None	3	1.80
	Don’t know	105	62.10
Mode of punishment for doctors	Fine	7	4.10
	Imprisonment	8	4.70
	Both	7	4.10
	None	4	2.40
	Don’t know	143	84.60
Mode of punishment for patients/relatives	Fine	7	4.10
	Imprisonment	7	4.10
	Both	4	2.40
	None	4	2.40
	Don’t know	147	87.0
Awareness and perceptions of PCPNDT Act [3]*			
Heard of government act against prenatal sex determination	Yes	26	15.40
	No	77	45.60
	Don’t know	66	39.10
Name of the act	Correct name	1	0.60
	Incorrect name	19	11.20
	Don’t know	149	88.20
Source of information	Doctors and health personnel	23	13.60
	Family and friends	5	3.0
	Media	31	18.30
	Other sources	1	0.60
	Don’t know	109	64.50
Opinion regarding punishment	Yes	99	58.60
	No	17	10.10
	Don’t know	53	31.40
Willingness to educate others about the PCPNDT Act	Yes	130	76.90
	No	2	1.20
	Don’t know	37	21.90
Gender preferences			
Preference to know sex of unborn child	Yes	47	27.80
	No	115	68.0
	Don’t know	7	4.14
Gender preference for current pregnancy	Boy	19	11.20
	Girl	43	25.40
	No preference	107	63.30
Ideal number of children	1	8	4.70
	2	42	24.90
	3	40	23.70
	4	45	26.60
	>4	34	20.10
Preferred gender composition overall	Only boys	1	6.0
	Only girls	5	3.0
	More boys than girls	5	3.0
	More girls than boys	10	5.90
	Equal number of boys and girls	108	63.90
	No preference	40	23.70

An assessment of multicollinearity and goodness-of-fit was conducted across all three multivariable logistic regression models. Collinearity testing confirmed no data redundancy, with all variable tolerances exceeding 0.20 and variance inflation factor (VIF) values below 5. While the model for awareness of prenatal sex determination (Hosmer-Lemeshow, p=0.143) showed adequate global fit, the variables combined did not create a statistically significant model (Omnibus, p=0.108). The model for awareness of the PCPNDT Act (Hosmer-Lemeshow, p=0.274; Omnibus, p=0.043) demonstrated a strong global fit and statistical significance of the model. The model for preference to know the sex of the unborn child showed a poor overall fit (Hosmer-Lemeshow, p=0.030), and the variables combined did not create a statistically significant model (Omnibus, p=0.91).

Variables such as age, education, occupation, socioeconomic status (SES) class, gestational age, and previous number of children were considered for a multivariate model assessing the factors associated with awareness of prenatal sex determination. Only education remained statistically significant and independently associated with awareness of prenatal sex determination. Pregnant women with higher than grade 12 education (AOR=4.12, 95% CI: 1.59-10.64, p=0.003) and grade 8-12 (AOR=2.45, 95% CI: 1.06-5.65, p=0.036) exhibited higher odds of knowing about the Act, when compared to those with education up to grade 7 or less (Table 3).

**Table 3:** Factors associated with awareness of prenatal sex determination and PCPNDT Act among study participants (n=169). *Adjusted odds ratio; 95% confidence intervals computed by multiple logistic regression. **P<0.05 was statistically significant. ***Socioeconomic status (SES) was assessed using the modified BG Prasad Socioeconomic Classification (2019) [7]. ****Pre-Conception and Pre-Natal Diagnostic Techniques (Prohibition of Sex Selection) Act [3]. PCPNDT: Pre-Conception and Pre-Natal Diagnostic Techniques

Factors associated with awareness of prenatal sex determination							
Variables		Aware of prenatal sex determination (n=71), n (%)	Unaware/don’t know about prenatal sex determination (n=98), n (%)	Total	AOR*	95% CI*	p-Value
Age (years)	30-40	20 (43.50)	26 (56.50)	46	1.89	0.81-4.36	0.138
	18-29	51 (41.50)	72 (58.50)	123	Reference		
Education	≥Grade 12	25 (55.60)	20 (44.40)	45	4.12	1.59-10.64	0.003**
	Grade 10-12	29 (44.60)	36 (55.40)	65	2.45	1.06-5.65	0.036**
	≤Grade 7	17 (28.80)	42 (71.20)	59	Reference	-	-
Occupation	Working	4 (44.40)	5 (55.60)	9	0.591	0.13-2.65	0.492
	Housewife	67 (41.90)	93 (58.10)	160	Reference		
SES [7]***	Class I+II	20 (40.0)	30 (60.0)	50	1.13	0.42-3.06	0.8
	Class III+IV	40 (44.40)	50 (55.60)	90	1.51	0.61-3.71	0.37
	Class V	11 (37.90)	18 (62.10)	29	Reference	-	-
Trimester	Third	30 (46.20)	35 (53.80)	65	1.33	0.68-2.59	0.4
	First and second	41 (39.40)	63 (60.60)	104	Reference		
Number of children	>2 children	5 (33.30)	10 (66.70)	15	0.81	0.21-3.11	0.76
	≤2 children	35 (41.20)	50 (58.80)	85	0.98	0.49-1.96	0.95
	Nil	31 (44.90)	38 (55.10)	69	Reference	-	-
Factors associated with awareness of the PCPNDT Act [3]****							
Variables		Aware of PCPNDT Act (n=26)	Unaware/don’t know of PCPNDT Act (n=143)	Total	AOR*	95% CI*	p-Value
Age (years)	30-40 years	11 (23.90)	35 (76.10)	46	6.682	1.99-22.43	0.002**
	18-29 years	15 (12.20)	108 (87.80)	123	Reference		
Education	≥Grade 12	14 (31.10)	31 (68.90)	45	24.49	4.61-130.2	<0.001**
	Grade 10-12	9 (13.80)	56 (86.20)	65	7.77	1.62-37.22	0.010**
	≤Grade 7	3 (5.10)	56 (94.90)	59	Reference	-	-
Occupation	Working	1 (11.10)	8 (88.90)	9	1.28	0.21-7.70	0.79
	Housewife	46 (28.70)	114 (71.30)	160	Reference		
SES****	Class I+II	7 (14.0)	43 (86.0)	50	1.12	0.25-4.97	0.88
	Class III+IV	15 (16.70)	75 (83.30)	90	1.61	0.42-6.12	0.49
	Class V	4 (13.80)	25 (86.20)	29	Reference	-	-
Trimester	Third	14 (13.50)	90 (86.50)	104	1.67	0.64-4.39	0.29
	First and second	12 (18.50)	53 (81.50)	65	Reference		

To explore the factors associated with awareness of the PCPNDT Act, the following variables were considered: age, education, occupation, SES class, and gestational age. After the multivariate analysis, age and higher education were statistically significant determinants of awareness. Older pregnant women aged 30-40 years had higher odds (AOR=6.68, 95% CI: 1.99-22.43, p=0.002) of being aware of the PCPNDT Act than younger pregnant women. Pregnant women with education higher than grade 12 (AOR=24.49, 95% CI: 4.61-130.2, p<0.001) and those with education up to grade 8-12 (AOR=7.77, 95%CI: 1.62-37.22, p=0.01) exhibited higher odds of knowing about the Act than did those with education up to grade 7. Variables such as occupation, SES class, and gestational age did not demonstrate a statistically significant impact on awareness about the PCPNDT Act (Table 3).

The association between the preference to know the sex of the unborn child and key sociodemographic and obstetric variables was explored. The following two factors emerged to be significantly associated: age and SES. Pregnant women in the age group of 18-24 years had about three times higher odds (AOR=3.12, 95% CI: 1.08-9.03, p=0.036) of having a preference to know the sex of the child than the reference group of 30-40 years. Pregnant women belonging to SES Classes III and IV had about three times higher odds (AOR=3.15, 95% CI: 1.26-7.88, p=0.014) of having a preference to know the sex of the unborn child than the reference group - Classes I and II (Table 4).

**Table 4:** Factors associated with preference to know the sex of the unborn child among study participants (n=169). *Adjusted odds ratio; 95% confidence intervals computed by multiple logistic regression. **P<0.05 was statistically significant. ***Socioeconomic status (SES) was assessed using the modified BG Prasad Socioeconomic Classification (2019) [7].

Variables		Prefer to know sex of unborn child (n=47), n (%)	No preference to know sex of unborn child (n=122), n (%)	Total	AOR*	95% CI*	p-Value
Age (years)	18-29	41 (33.30)	82 (66.70)	123	3.12	1.08-9.03	0.036**
	30-40	6 (13.0)	40 (87.0)	46	Reference		
Education	≤Grade 7	13 (22.0)	46 (78.0)	59	0.834	0.306-2.78	0.72
	Grade 10-12	20 (30.80)	45 (69.20)	65	0.824	0.34-2.01	0.67
	≥Grade 12	14 (31.10)	31 (68.90)	45	Reference		
Occupation	Housewife	46 (28.70)	114 (71.30)	160	3.106	0.34-28.32	0.31
	Working	1 (11.10)	8 (88.90)	9	Reference		
SES [7]***	Class V	19 (18.30)	10 (15.40)	29	2.48	0.80-7.71	0.12
	Class III+IV	61 (58.70)	29 (44.60)	90	3.15	1.26-7.88	0.014**
	Class I+II	24 (23.10)	26 (40.0)	50	Reference		
Trimester	Third	28 (26.90)	76 (73.10)	104	1.23	0.59-2.58	0.58
	First and second	19 (29.20)	46 (70.80)	65	Reference		
Number of children	Nil	21 (30.40)	48 (69.60)	69	1.59	0.28-8.99	0.59
	≤2 children	24 (28.20)	61 (71.80)	85	1.76	0.33-9.34	0.51
	>2 children	2 (13.30)	13 (86.70)	15	Reference		
Gender of last child	Boy	15 (27.80)	39 (72.20)	54	0.94	0.43-2.08	0.88
	Girl	12 (26.10)	34 (73.90)	46	0.86	0.37-2.01	0.73
	Not applicable	20 (29.0)	49 (71.0)	69	Reference		

## Discussion

It has been reported that the implementation of the PCPNDT Act in India has not achieved the desired success. It has also been observed that there exists a strong preference for male children in Indian society, despite socioeconomic development and advancing growth rates [7-9]. To gain further insights on these issues, the present study was carried out with the primary objective of determining the awareness and perceptions of prenatal sex determination, the PCPNDT Act, and gender preferences of the unborn child among pregnant women.

In our study, the majority of the respondents belonged to the 18-29 years age group. Moreover, half of them belonged to the middle class and lower-middle class (Class III and IV). A study conducted in a teaching hospital in Mumbai (Shidhaye et al.) also reported that 73.40% of the women were in the age group of 20-30 years, while 42.60% belonged to the lower-middle class [9,10]. Another study by Larsson et al. included participants aged 25-34 years [11].

Awareness about the possibility of prenatal sex determination was present in 42.0% of the respondents. This finding was strikingly similar to the results of a study carried out in Darjeeling, which reported that 44.82% were aware of prenatal sex determination (Roy and Biswas) [12]. On the contrary, a Mumbai study by Shidhaye et al. reported that 73.50% of antenatal women were aware that prenatal sex determination can be carried out [9]. Another study conducted in Mangalore (Kumar et al.) reported that a high proportion, i.e., 91.10%, were aware of prenatal sex determination. Further, the authors of that study also attributed this finding to a high literacy rate in Mangalore [4]. It is important to note here that nearly three-fourths of respondents in our study were educated only up to grade 10. In fact, a mere 9.50% were graduates.

A low proportion mentioned that ultrasound could be used as a method of prenatal sex determination. Similarly, a study by Sharmila et al. in Puducherry reported that 87.50% mentioned ultrasound as a method for prenatal sex determination [5]. However, in a study done in Nagpur, 30.0% of the mothers of under-five children enlisted ultrasonography as a prenatal sex determination technique [13].

Furthermore, in our study, a low proportion (15.0%) of antenatal women were aware that there exists a government act against prenatal sex determination. Contrary to this, a study carried out in Puducherry reported that 68.0% of antenatal women were aware of a government act to curb prenatal sex determination (Sharmila et al.) [5].

Higher levels of education were associated with awareness about prenatal sex determination and the PCPNDT Act. Individuals with lower education often experience lower health literacy and may have barriers to antenatal counseling sessions. This subgroup is more likely to rely on localized, peer-to-peer information for health-related affairs and is less likely to consume digital health media. Looking through the lens of the social determinants of health framework, the educational background of pregnant women shapes their sociocultural position, beliefs, and value system. Women with lower education often have limited autonomy over reproductive choices and health-seeking behavior. This may facilitate the information vacuum, preventing medical and legal aspects around prenatal sex determination from reaching women with lower education.

The diagnostic checks for regression model revealed that only the model for awareness of the PCPNDT Act achieved statistical significance with an adequate data fit, but the magnitude of the AOR must be interpreted with caution, as the 95% confidence intervals are likely artificially inflated due to small subgroup sizes and an underpowered sample size. The remaining two models, awareness of prenatal sex determination and preference to know the sex of the unborn child, lacked overall significance, and the AORs must be interpreted as exploratory findings.

Lower SES categories had a significant association with preference to know the sex before birth. Similarly, in another study conducted in Eastern India, gender preference was significantly higher in subjects with lower socioeconomic status (p=0.011) [9,10]. This association may likely stem from sociocultural norms favoring a particular gender or from a desire to better prepare for cultural milestones of the child in resource-constrained households.

The strength of this study is that it was able to provide a distinct insight into how aware pregnant women are regarding prenatal sex determination practices, the PCPNDT Act, and also their preference for the unborn child’s sex. Another added strength is that we were also able to capture quality-assured data with the use of Epicollect5 through a smartphone, which minimized the errors that are usually encountered during data collection and entry.

Limitations in the methodology include convenience sampling in a hospital-based setting and selection bias, which limit the generalizability of the study findings to the general population, although providing a valuable baseline for this study setting. The findings related to gender preferences of the unborn child among the respondents need to be cautiously interpreted. Due to the presence of certain questions related to punishment for prenatal sex determination and features of the PCPNDT Act, there could be a possibility of social desirability bias, in which the participants may have concealed their preference for the sex of their unborn child. The study sample size was not powered to assess factors associated with awareness of prenatal sex determination, the PCPNDT Act, and gender preference; therefore, the results must be interpreted with caution.

## Conclusions

In this study, a substantial proportion of pregnant women did not have baseline awareness regarding the legal prohibition against prenatal sex determination. More than one-third had a gender preference for the unborn child. This study reflects the need to educate antenatal women and their families about the ethical and legal implications of prenatal sex determination and the salient features of the PCPNDT Act. These findings warrant the promotion of a gender-neutral attitude among pregnant women and their families. There is further scope for research to determine the proportion of antenatal women who have been sensitized by doctors or other healthcare professionals regarding the PCPNDT Act.

## References

[1] Kanitkar T, Mistry M (2000). Status of women in India: an interstate comparison. Indian J Soc Work.

[2] Census of India 2011: population by age and sex, and sex ratio. https://censusindia.gov.in/census.website/data/data-visualizations/Age-Gender-Ratio_Pyramid-Chart.

[3] Khanna V, Gopal KM (2024). Pre-Conception and Pre-Natal Diagnostic Techniques Act - draconian or a considerate de jure tamer. Int J Reprod Contracept Obstet Gynecol.

[4] Kumar N, Darshan BB, Unnikrishnan B (2014). Awareness and attitudes regarding prenatal sex determination, Pre-Conception and Pre-Natal Diagnostic Techniques Act (PCPNDTA) among pregnant women in Southern India. J Clin Diagn Res.

[5] Sharmila V, Babu TA, Singh D (2016). Knowledge, awareness and attitude about prenatal sex determination, Pre-Conception and Pre-Natal Diagnostic Techniques Act among pregnant women in the South Indian union territory of Puducherry. Int J Reprod Contracept Obstet Gynecol.

[6] Nithin K, Tanuj K, Unnikrishnan B (2015). Gender preferences among antenatal women: a cross-sectional study from coastal South India. Afr Health Sci.

[7] Pandey VK, Aggarwal P, Kakkar R (2019). Modified BG Prasad Socio-economic Classification, update - 2019. Indian J Community Health.

[8] Tabaie S (2017). Stopping female feticide in India: the failure and unintended consequence of ultrasound restriction. J Glob Health.

[9] Shidhaye PR, Giri PA, Nagaonkar SN, Shidhaye RR (2012). Study of knowledge and attitude regarding prenatal diagnostic techniques act among the pregnant women at a tertiary care teaching hospital in Mumbai. J Educ Health Promot.

[10] Yasmin S, Mukherjee A, Manna N (2013). Gender preference and awareness regarding sex determination among antenatal mothers attending a medical college of eastern India. Scand J Public Health.

[11] Larsson M, Berglund M, Jarl E, Tydén T (2017). Do pregnant women want to know the sex of the expected child at routine ultrasound and are they interested in sex selection?. Ups J Med Sci.

[12] Roy A, Biswas R (2017). A study on gender preference and awareness regarding prenatal sex determination among antenatal women in a rural area of Darjeeling district, West Bengal, India. J Clin Diagn Res.

[13] Rawat S, Yadav A, Bhate K (2021). Gender determination and gender gap: a cross sectional comparative study of mothers attending under five immunisation clinics in urban and rural areas. J Family Med Prim Care.

